# Supplementary material for: Crack-Based Sensor by Using the UV Curable Polyurethane-Acrylate Coated Film with V-Groove Arrays
Source: Micromachines (Basel). 2022 Dec 26;14(1):62. doi: 10.3390/mi14010062 (PMC9862563; doi:10.3390/mi14010062)
Supplement: Supplementary file 1 [file micromachines-14-00062-s001.zip › micromachines-2093898-supplementary.pdf]

Supplementary Information

For

Crack-Based Sensor by using the UV Curable Polyure-thane-acrylate Coated Film with V-groove Arrays

The strain applied to the sensor was calculated using equation reported in the previous manuscript [27]. We employed the bent radius and the central q factor when the tension strain applied on the sensor.

As our device, Under tensile mode,

$$l = \theta(r + h)$$

$$c = 2r \cdot \sin\left(\frac{\theta}{2}\right)$$

Therefore, the tension strain $\Delta\varepsilon$ can be described as follows:

$$\Delta\varepsilon = \frac{\Delta l}{l} = \frac{l_1 - l_0}{l_0} = \frac{\theta_1(r_1 - z) - \theta_0(r_0 - z)}{\theta_0(r_0 - z)}$$

Since, the equation can be converted to when the $r_1 \gg z,\ \theta_1 r_1 \approx \theta_0 r_0$

$$\Delta\varepsilon = \frac{\Delta l}{l} = \frac{z(\theta_1 - \theta_{0)}}{r_0 l_0} = \frac{z}{r_0}\left(\frac{\theta_1}{\theta_0} - 1\right) \approx \frac{z}{r_0}\left(\frac{r_1}{r_0} - 1\right) = z\left(\frac{1}{r_1} - \frac{1}{r_0}\right)$$

Since r_0 tend to infinity

$$\Delta\varepsilon = -\frac{z}{r_1} = \frac{h}{2r_1}$$

27. Ham, Z.; Liu, L.; Zhang, J.; Han, Q.; Wang, K.; Song, H.; Wang, Z.; Jiao, Z.; Niu, S.; Ren, L. High-performance flexible strain sensor with bio-inspired crack arrays. *Nanoscale* **2018**, *10*, 15178–15186.
